# Effect of Processing on Bioactive Compounds, Antioxidant Activity, Physicochemical, and Sensory Properties of Orange Sweet Potato, Red Rice, and Their Application for Flake Products

**DOI:** 10.3390/plants11030440

**Published:** 2022-02-05

**Authors:** Ignasius Radix A. P. Jati, Laurensia M. Y. D. Darmoatmodjo, Thomas I. P. Suseno, Susana Ristiarini, Condro Wibowo

**Affiliations:** 1 Department of Food Technology, Widya Mandala Surabaya Catholic University, Jl. Dinoyo 42-44, Surabaya 60265, Indonesia; yulian@ukwms.ac.id (L.M.Y.D.D.); thomasindartoftp@gmail.com (T.I.P.S.); ristiarini@ukwms.ac.id (S.R.); 2 Department of Food Technology, Jenderal Soedirman University, Jl. Dr. Soeparno 61, Purwokerto 53123, Indonesia; condro.wibowo@unsoed.ac.id

**Keywords:** orange sweet potato, red rice, flakes, bioactive compound, antioxidant activity, physicochemical, sensory properties

## Abstract

Orange sweet potato (OSP) and red rice (RR) are rich sources of health benefit-associated substances and can be conventionally cooked or developed into food products. This research approach was to closely monitor the changes of bioactive compounds and their ability as antioxidants from the native form to the food products which are ready to be consumed. Moreover, this research explored the individual carotenoids and tocopherols of raw and cooked OSP and RR and their developed flake products, and also investigated their antioxidant activity, physicochemical properties, and sensory properties. Simultaneous identification using the liquid chromatographic method showed that OSP, RR, and their flake products have significant amounts (µg/g) of β-carotene (278.58–48.83), α-carotene (19.57–15.66), β-cryptoxanthin (4.83–2.97), α-tocopherol (57.65–18.31), and also γ-tocopherol (40.11–12.15). Different responses were observed on the bioactive compound and antioxidant activity affected by heating process. Meanwhile, OSP and RR can be combined to form promising flake products, as shown from the physicochemical analysis such as moisture (5.71–4.25%) and dietary fiber (13.86–9.47%) contents, water absorption index (1.69–1.06), fracturability (8.48–2.27), crispness (3.9–1.5), and color. Those quality parameters were affected by the proportions of OSP and RR in the flake products. Moreover, the preference scores (n = 120 panelists) for the flakes ranged from slightly liked to indifferent. It can be concluded that OSP and RR are potential sources of bioactive compounds which could act as antioxidants and could be developed into flake products that meet the dietary and sensory needs of consumers.

## 1. Introduction

Modern food trends and lifestyle changes have strongly influenced the dietary habits of society. The demands for ready-to-eat and simple-to-prepare foods are increasing rapidly, providing an excellent opportunity for food industries to play a significant role in supplying such food products. Flakes, one of the most popular foods made from cereals, typically oat, corn, and barley, are commonly served for breakfast with milk in Europe and the USA, and their global appeal is gaining traction. In addition, healthy eating has become a new trend in modern culture, with consumers increasingly opting for healthy food options. Secondary metabolites found in plants have been shown to decrease the risks of degenerative diseases such as coronary heart disease, diabetes, cancer, and stroke [1,2,3,4]. Thus, innovative functional food products rich in bioactive compounds are needed to promote a healthy diet and reduce disease risks. Asia has the most rapidly growing food product market, so using local ingredients can help open a new market for flakes and decrease the reliance on imported foods such as oat and barley. Commodities that can potentially be developed into flakes are red rice (RR) and orange sweet potato (OSP).

RR (*Oryza nivara* L.) is a variety of rice with red pericarp caused by anthocyanins in the aleurone layer. It is a rich source of anthocyanins such as cyanidin 3-O-glucoside and peonidin 3-O-glucoside [2]. Anthocyanins have also been reported to inhibit plaque formation [3] and to exhibit hypocholesterolemic [4] and anticancer effects [5] in RR. The health benefits of RR have also been linked to bioactive compounds such as tocopherol and tocotrienols [6,7] and dietary fiber [8]. RR also contains higher essential minerals than white rice, including iron, zinc, and vitamins, which are especially important for babies and toddlers, [9]. Despite the various health benefits of RR, its consumption remains low. It is generally considered inferior to white rice due to the hard texture and unpleasant aroma when cooked. Traditionally, RR is consumed steamed or boiled, and its sensory properties are inferior to white rice. The most popular RR-based product is RR baby porridge, but there are reports on the development of RR-based products such as pasta [10], noodles [11], flakes [12], rice milk [13], and fermented beverages [14]. However, these have not been scaled up or commercially established.

OSP (*Ipomoea batatas*) is one variety of sweet potato with a bright orange flesh color caused by carotenoids, of which high amounts of β-carotene, α-carotene, and β-cryptoxanthin have been reported [15,16,17]. In many countries, OSP has been used to eradicate vitamin A deficiency due to its high content of beta-carotene, a pro-vitamin A carotenoid. Sweet potato was extensively promoted in Africa and some Asian countries with remarkable results [18,19,20]. However, there were difficulties in ensuring its sustainability due to the monotonous method of preparation, mainly boiled and baked, even though the essential nutritional components were reportedly retained after processing [21]. Processing OSP could decrease the beta-carotene, but not below the recommended dietary level [22]. Moreover, food prepared from OSP by the traditional method was not attractive to children, who were the main target of the vitamin A intake enhancement. Therefore, innovative food products need to be developed to promote the consumption of RR and OSP. Numerous research has been published to explore the potency of various plant sources as functional foods. However, the approach mainly investigates the plant materials in the native or raw form. In contrast, the consumer will generally consume after the materials undergo transformations which could affect the characteristics of the products [23]. Moreover, besides the processing, the bioactive compounds and antioxidant activity will be further affected by the in vivo digestion and the intestinal absorption rate of the body metabolism before providing bioavailable compounds that can be used [24]. This research approach was to closely monitor the changes of the bioactive compound and antioxidant activity from the raw materials to the ready to be consumed food products, and aimed to investigate the bioactive compounds and antioxidant activity of raw and cooked RR and OSP and the physicochemical and sensory properties of their developed flake products.

## 2. Materials and Methods

### 2.1. Plant Materials and Chemicals

A local variety of RR (*Oryza nivara* L.), “Cempo abang”, and OSP (*Ipomoea batatas* L.), “Mendut”, were collected from farmers in Yogyakarta province, Indonesia. Chemicals used for analysis, including distilled water, Folin–Ciocalteu, 1,1-diphenyl-2-picrylhydrazyl (DPPH), gallic acid, butylated hydroxyl toluene (BHT), enzymes (thermamyl, pancreatin, pepsin), riboflavin, methionine, and nitroblue tetrazolium (NBT), were purchased from Sigma Chemical. Methanol, Whatman 40 filter paper, n-hexane, NaOH, HCl, ethanol, and phosphate buffer (pH 6) were purchased from Merck, Germany. Carotenoid standards, including β- and α-carotene, β-cryptoxanthin, lycopene, and lutein, and α- and γ-tocopherol standards were obtained from Sigma-Aldrich.

### 2.2. Sample Preparation

#### 2.2.1. Raw Samples

The RR samples were washed, drained, and blended (Philips food processor). The OSP samples were chopped into small pieces. All samples were then freeze-dried, refined, and sieved (30 mesh). Finally, the powdered samples were placed in dark bottles and stored in a refrigerator (4 °C) for further usage.

#### 2.2.2. Boiled Samples

RR (75 g) was cooked with 135 g of tap water (1:1.8; *w*/*w*) in a rice cooker (Panasonic) for approximately 45 min. After, the cooked rice was cooled for 10 min. Meanwhile, 150 g of OSP was boiled in an aluminum pot using tap water for 20 min, cooled for 10 min, and then mashed. Both samples were freeze-dried, refined, sieved (30 mesh), and stored (4 °C) in a refrigerator.

#### 2.2.3. Flakes Production

RR was placed in a cabinet dryer (60 °C) for 1 h. The OSP was peeled, sliced and placed in a cabinet dryer (60 °C) for 6 h. The dried OSP and RR were mashed using a blender. The flour was passed through an 80 mesh. Flakes were produced using six different proportions of OSP and RR, namely 100:0, 80:20, 60:40, 40:60, 20:80, and 0:100. Salt (3% *w*/*w*), sugar (30% *w*/*w*), and water (150% *w*/*w*) were mixed in as additional ingredients. The mixture was heated at 75 °C for 1 min and pressed at 170 °C for 1 min using a customized flake pressing tool. The pressed flakes were cut at 2 × 2 cm and dried using an oven at 125 °C for 5 min.

### 2.3. Bioactive Compounds and Antioxidant Activity Analysis

#### 2.3.1. Methanolic Extract of Samples

The extraction of samples (raw, boiled, and flakes) was performed according to a previously published procedure [25]. Briefly, 1 g of sample was weighed, ground, placed in a centrifuge tube, and then extracted with 10 mL of 1% methanol–HCl solution. The mixture was then vortexed for 15 min, centrifuged at 5000 rpm for 15 min, filtered (Whatman No. 40), and then used for antioxidant activity analysis, performed in triplicate.

#### 2.3.2. Phenolic Content

The total phenolic content was determined using the Folin–Ciocalteu method by Singleton and Rossi as described in other published work [26]. In brief, 0.1 mL of extract was mixed with 0.5 mL 1:1 Folin–Ciocalteu reagent and distilled water. After 10 min, 4.5 mL of 2% sodium carbonate (Na_2_CO_3_) was added, and the mixture was then vortexed and kept in the dark for 1 h. The blue complex formed was measured using a spectrophotometer at 765 nm. Methanol and gallic acid were used as the blank and standard, respectively. The results were calculated as milligram gallic acid equivalents (GAE)/100 g dry weight. 

#### 2.3.3. Anthocyanin Content

The total anthocyanin in RR and flake samples was determined spectrophotometrically using the pH differential method [27]. In brief, 1 mL of extract was diluted in pH 1.0 and pH 4.5 buffers. The absorbance was measured at 510 and 710 nm. The final absorbance was calculated using the formula:

A = [(A513-A700)pH 1.0 − (A513-A700)pH 4.5)](1)

The calculated absorbance was then used to calculate the total grams of anthocyanins per 100 g dry weight, with a molar extinction coefficient of 26,900 and a molecular weight of 445. 

#### 2.3.4. Carotenoid and Tocopherol Analysis

The carotenoids and tocopherols in the raw and boiled samples of RR and OSP were determined simultaneously using High-Performance Liquid Chromatography (HPLC) based on previously published research [24]. In brief, 0.3 g of finely ground samples were extracted with 0.5 mL of 70% ethanol and 0.4 mL of n-hexane under yellow lights. In addition, 0.4 mL β-Apo-8’carotenal-O-methyloxim and 0.4 mL α-, γ-tocopherol were used as an internal standard for carotenoids and tocopherols, respectively. The mixture was shaken for 20 min, centrifuged at 5000 rpm, 4 °C for 20 min. Next, the upper layer of extract containing a hexane fraction was collected using a micropipette. The extraction was repeated four times using only n-hexane as a solvent. Finally, the hexane fractions were pooled and completely dried using nitrogen gas. 

Before injection, the extracts were mixed with 200 µL of ethanol containing 30 µg/mL BHT, then 20 µL of the mixture was injected into the HPLC (Varian Pro Star 410, Spark, The Netherlands). A mixture of 82% acetonitrile, 15% dioxan, 3% methanol, 0.1 M ammonium acetate, and 0.1% triethylamine was assigned as the mobile phase and was pumped at a rate of 1.6 mL/min. The solvent was pre-mixed to avoid dependency on reproducible mixing by the pump. For separation, a C18 Spherisorb ODS 2 column (3 µm, 250 × 4.6 mm) was applied. In addition, a UV Vis detector at 450 nm and a Scanning Fluorescence detector using an excitation wavelength of 295 nm and an emission wavelength of 328 nm were used to monitor the carotenoids and tocopherols, respectively. Five repetitions were performed for the HPLC analysis.

#### 2.3.5. DPPH Radical Scavenging Activity

The radical scavenging activity of the extract was examined by the DPPH method [25]. In brief, 1 mL of extract was mixed with 2 mL of 0.2 M DPPH and 2 mL of methanol in centrifuge tubes, vortexed, and kept in the dark for 1 h. The absorbance was measured spectrophotometrically at 517 nm. As a control, 150 ppm BHT solution was used. The DPPH radical scavenging activity of the extract was expressed as a percentage calculated as follows: % radical scavenging capacity = ((Absorbance of control – Absorbance of sample)/Absorbance of control) × 100%

#### 2.3.6. FRAP Assay

The ferric reducing antioxidant power (FRAP) was examined based on a previously published report [28]. In brief, a mixture of 60 µL extract, 180 µL distilled water, and 1.8 mL FRAP reagent was vortexed and incubated at 37 °C for 30 min. The spectrophotometer was used to read the absorbance of the mixture at 593 nm. A standard curve was prepared with Fe [II] (FeSO_4_.7H_2_O, 100–2000 mM) to calculate the reducing power. The result was expressed as mmol Fe[II]/g. In addition, methanol was used for the reagent blank.

#### 2.3.7. Superoxide Radical Scavenging Capacity

A previous report [29] was followed to examine the Superoxide radical scavenging capacity. Firstly, a reagent containing riboflavin, methionine, and NBT in 0.05 M phosphate buffer pH 7.8 was prepared. Then, 100 µL of the extract was mixed with 4.9 mL of reagent and illuminated (20 W fluorescent lamp) at 25 °C for 25 min. The absorbance was measured at 560 nm.

### 2.4. Physicochemical Properties of Flake Products

#### 2.4.1. Moisture and Dietary Fiber Contents 

The moisture content was measured thermogravimetrically [30]. In brief, 1 g of each sample was dried at 105 ± 0.2 °C to establish a constant mass. The analysis was performed in triplicate. 

#### 2.4.2. Water Absorption Index

The water absorption index was examined according to previously published method [31]. In brief, 5 g of flakes was placed in a 100 mL beaker, 30 mL of water at 30 °C was added. After 10 s of immersion, the flakes were dried. The water absorption index was calculated using the formula: WAI = (wf -wi)/wi, where wi and wf are the initial and final weight of the sample, respectively.

#### 2.4.3. Fracturability, Crispness, and Color Profiles

The fracturability and crispness of flakes were measured using TA-XT Plus Texture Analyzer (Stable Micro Systems, Surrey, UK) [32]. The probe used was a ¼ inch spherical stainless-steel probe (P0.25S). The sample was placed on the sample holder, and then the probe was moved down to press the sample. The results were obtained in the form of a graph (force vs. time) (the graph is not shown). The value of the *y*-axis at the graph’s highest point is the maximum force value that can be held by the sample, called the value of fracturability. Crispness can be measured through changes in displacement distance during a drastic decline in the graph pattern from the highest peak to the next peak point. Meanwhile, the color profiles of flakes were measured using color reader Konica Minolta CR-10 (Konica Minolta, Osaka, Japan). The results were expressed as Lightness (L*), redness (a*), yellowness (b*), ^o^hue (^o^h), and Chroma (C).

### 2.5. Sensory Analysis

The sensory evaluation was conducted by 120 untrained panelists to determine the level of consumer preference for the flakes with various proportions of OSP and RR. The parameters tested were preferences for color, taste, crispness of flakes, and mouthfeel when served with milk. The Hedonic Scale Scoring method (preference test) with a scale ranging from 1 (strongly disliked) to 7 (strongly liked) was used for the sensory test. 

Samples of flakes for the color preference test were prepared in open white plastic containers. Panelists were asked first to assess aspects of flakes’ taste, color, and crispness before serving with milk. The crispness was evaluated based on the panelist’s preference for the sound of flakes during biting. For the mouthfeel test, 5 g of flakes were prepared in a small plastic container. Panelists were instructed to pour 10 mL of milk into the container and wait for 1 min. Then, they were asked to assess the mouthfeel of the flakes based on preference level by filling the questionnaire sheet provided. 

### 2.6. Statistical Analysis

The data were statistically analyzed using ANOVA (α = 5%) followed by Duncan’s Multiple Range Test (DMRT) on SPSS software version 19. Spider web chart analysis using Microsoft Excel was used to determine the best proportion of OSP and RR in flakes based on the panelists’ preferences.

## 3. Results

### 3.1. Bioactive Compounds of OSP, RR, and Their Flake Products

Table 1 shows the phenolic compound, anthocyanin, carotenoid, and tocopherol contents of raw and cooked OSP and RR and their flakes containing different proportions of OSP and RR. The raw RR had a higher phenolic content than OSP. As a result, the higher the proportion of RR, the higher the phenolic content of the developed flakes. It was also found that cooking decreased the phenolic content of RR and OSP by approximately 49.34% and 41.08%, respectively.

Interestingly, the flakes with 100% RR showed a higher phenolic content than the cooked RR. Anthocyanin was only observed in RR. Furthermore, flakes containing 100% RR had a lower anthocyanin content than the conventionally cooked RR.

Of the carotenoids, OSP had a higher content of β-carotene, α-carotene, β-cryptoxanthin, and lutein. β-carotene and β-cryptoxanthin were the dominant carotenoids observed in RR. The cooking process significantly decreased the content of β-carotene in OSP and RR by roughly 48% and 56%, respectively. Overall, flakes containing higher amounts of OSP showed higher carotenoid contents. The processing significantly decreased the carotenoids in the flake products when considering the raw forms and the proportions of OSP and RR, and β-carotene was the major carotenoid remaining in the flake products.

Moreover, raw RR contained higher α-tocopherol than OSP in raw forms. Unlike the decreasing trend observed in other bioactive compounds due to processing, the tocopherol and α-carotene contents of both RR and OSP increased after conventional cooking. On the other hand, the two-step thermal processing decreased the tocopherol content of flakes.

### 3.2. Antioxidant Activity of Raw and Cooked OSP and RR and Their Flake Products

**Figure 1:**
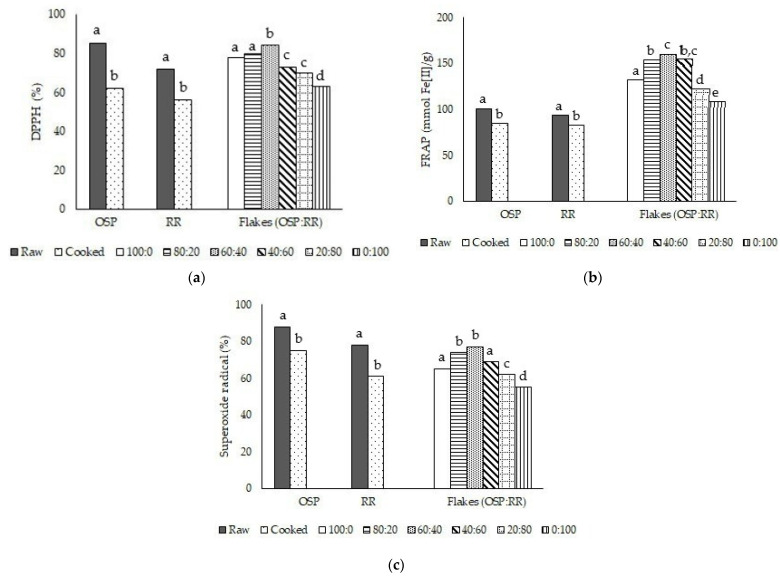
Antioxidant activity of extract determined by (**a**) DPPH, (**b**) FRAP, and (**c**) Superoxide radical assays. Different superscript letters (a–e) denote significantly different values according to Duncan’s test (*p* < 0.05). Comparison was made within each category (OSP: orange sweet potato, RR: red rice, and flakes).

The antioxidant activity of raw and cooked OSP and RR and their flake products were examined using DPPH, FRAP, and Superoxide radical scavenging activity methods. Figure 1a shows the DPPH scavenging activity of methanolic extract of RR, OSP, and the flake products. Boiling affected the ability of the methanolic extract to scavenge DPPH radicals. Approximately 16% and 23% decreases were observed in cooked RR and OSP, respectively. The combination of OSP and RR in the ratio of 60:40 resulted in flakes with the highest antioxidant activity (84%). The results trend indicated that the higher proportion of OSP contributed to the more robust antioxidant capacity of the extract. Furthermore, the DPPH result was in agreement with FRAP (Figure 1b) and Superoxide scavenging capacity (Figure 1c). Thus, conventional cooking and flake processing methods reduce the antioxidant activity of extracts of OSP and RR compared to their raw forms, and the right combination of OSP and RR in the flake formulation is critical for higher antioxidant activity.

### 3.3. Physicochemical Properties of OSP- and RR-Based Flake Products

The proportion of OSP and RR in the flake formulation affected the moisture content of flakes. A lower OSP proportion resulted in a lower moisture content of flakes (Table 2). The dietary fiber content increased with a higher proportion of RR. The dietary fiber content of flake products ranged from 9.47 ± 0.01% to 13.86 ± 0.73%.

The water absorption index of flakes was lowest at an OSP to RR ratio of 40:60, with 0.96 ± 0.03%, and was generally higher at combination ratios of 100:0, 80:20, 0:100, and 20:80. In addition, the texture characteristic of flakes was determined by the fracturability and crispness. The highest fracturability value was found in flakes made from 100% OSP (8.48 ± 0.09). The reduction of the OSP proportion in flakes caused a decrease in fracturability until the proportion of 40:60 (2.27 ± 0.04), beyond which the fracturability of flakes increased. A similar trend was observed in the crispness value of flakes. The lowest crispness value was detected in flakes with an OSP to RR ratio of 60:40, while higher values were obtained at ratios of 100:0, 0:100, 20:80, and 80:20.

Furthermore, the color of flakes was affected by the color of OSP and RR. The color profile of the flakes is shown in Table 3. 

### 3.4. Sensory Characteristics of Flakes

A preference test was conducted to determine the sensory characteristics of the flakes. The results are presented in Table 4. The highest level of color preference was found in flakes with OSP to RR ratios of 60:40, 40:60, and 20:80, while flakes containing 100% OSP had the lowest level of acceptance for color. The preference scores for taste and crispness of the flakes with various proportions of OSP and RR were comparable. Flakes containing 100% OSP and 100% RR received the highest preference score for mouthfeel, with the former having a significant edge. Therefore, mixing the OSP and RR lowers the mouthfeel acceptance of the flake products.

## 4. Discussion

Phenolic compounds are the most widely found bioactive compounds in plants [33]. Some are produced in response to stress conditions as a defense mechanism of the plant. They have been extensively investigated due to their antioxidant activity and anti-degenerative disease effects [34]. In this research, methanol–HCl (1%) was used because acidic methanol can penetrate deeply into cells, disrupting the cell membrane. In addition, acidic methanol can dissolve and stabilize polar compounds such as phenolics and anthocyanins [35]. This research shows that RR and OSP are rich sources of phenolic compounds. It has previously been reported that RR has a high content of phenolic compounds [36], and that these compounds are primarily accumulated in the aleurone layer and bran of RR [37]. Thus, the rice milling process to remove the husk plays a vital role in preventing the loss of various beneficial compounds. Previously, it was suggested that ferulic acid, p-coumaric acid, and pro-catechuic acid are the most abundantly found phenolic compounds in RR [37].

Here, cooking led to a 49% decrease in the phenolic compounds of RR. Heating of RR generally destroys the structure of phenolic compounds by breaking the esterified and glycosylated bonds, thus decreasing the quantified content of phenolic compounds in cooked RR [38]. Our results agree with previous findings [39,40], which reported high levels of total phenolic compounds, mostly gallic acid, chlorogenic acid, pro-catechuic 4-hydroxybenzoic acid, and salicylic acid, in different varieties of OSP. However, phenolic contents were broken down by the heating process. Regarding the flake products, flakes containing a higher proportion of RR showed a higher content of phenolic compounds. Nevertheless, the processing lowered the phenolic compounds in flakes when compared to raw OSP and RR. Boiling and baking have previously been reported to be responsible for the loss of phenolic compounds of RR-based products [41]

A similar trend was observed in the anthocyanin content of RR. The cooking process resulted in a significant decrease in the anthocyanin content due to the unstable property of anthocyanins when exposed to high temperatures [42]. Anthocyanins were only detected in RR in this research. Anthocyanins such as cyanidin 3 glucoside, delphinidin 3 glucoside, and peonidin are reported to have health-promoting properties [43]. Thus, exposure to high temperatures for extended periods should be avoided to reduce the risk of anthocyanin breakdown. The anthocyanin content in flakes was lower than in the raw samples, and increasing the proportion of RR resulted in a higher anthocyanin content of flakes. The decrease in the anthocyanin content of flakes is possibly due to the heat treatment during flake production, typically involving two high temperature processing steps of pre-gelatinization and flaking. It has been reported that the high-temperature treatment used for food processing can lead to a reduction of the anthocyanin content [44].

Moreover, there is a growing research interest in the conversion of β-carotene and α-carotene absorbed in the duodenum to retinol by intestinal enzymes [45]. The high rate of vitamin A deficiency in the world and the detrimental effects caused by the condition have necessitated the search for foods that can supply sufficient amounts of daily vitamin A requirement. In this research, OSP had the highest content of β-carotene. However, boiling of OSP decreased the β-carotene by approximately 41% of the β-carotene available. This phenomenon could be due to the thermal breakdown of β-carotene. Moreover, carotenoids are well known as substances that are sensitive to light and high temperatures [46]. RR contains different types of carotenoids [47]. Here, an increase in carotenoids after boiling was found, which could be related to the thermal disruption of the protein–carotenoid complex and the consequent release of carotenoids from the matrix. Similar findings have been published [48]. Similar trends were also observed in the OSP- and RR-based flake products. The β-carotene was significantly lower in the cooked sample compared to the raw sample. The OSP proportion in the flake formulation affected the carotenoid content. The higher the OSP proportion, the greater the carotenoid content of the flakes. In addition, the carotenoid contents of the flakes were significantly lower than the raw material. The simultaneous heating process from pre-gelatinization to flake pressing could further break down the carotenoids. This finding is supported by previously published work, which shows that heat treatment during food processing is responsible for the loss of carotenoids to degradation [49].

Vitamin E deficiency could lead to severe neurological problems. The main vitamin E compounds are tocopherols, with both α- and γ-tocopherol providing most vitamin E activity. Both samples had high contents of α- and γ-tocopherol. A significant increase (74%) in α-tocopherol was found in OSP after boiling. This result indicates that heat treatment could be beneficial for the bioaccessibility of tocopherol. Furthermore, heat treatment can assist in the breakdown of complex foods. Thus, tocopherol can be quickly released from its binding site. On the contrary, heat treatment was reported to reduce the tocopherol content in corn [50]. The increase in tocopherol after boiling indicates that tocopherols in the sample are more stable to heat treatment than other foods. In this research, RR had a considerably high tocopherol content. Therefore, boiling could have released the tocopherols from their binding site, facilitating their extraction and detection. Moreover, the structure of the rice grains could have played a role. RR has a compact structure. Therefore, cooking could assist the extraction of tocopherols, increasing the extractable tocopherol content, although some of the tocopherols might be lost to high temperature. The tocopherol contents of the different flake products were lower than the raw and boiled OSP and RR. It could be due to the simultaneous or prolonged exposure to heat treatment. Unlike the conventional method of cooking, which increases the tocopherol content, extended heating breaks the matrix structure in flakes and significantly affects the tocopherols. Thus, prolonged heating should be avoided in the processing of healthy food products to retain their tocopherols. Heat treatment combined with mechanical treatment in specific conditions could release the tocopherols from the food matrix. However, extended exposure will lead to the breakdown of carotenoids in the sample.

This research measured the antioxidant activity of raw and cooked OSP and RR and their flake products using DPPH, FRAP, and Superoxide radical scavenging capacity assays. The result showed that heat treatment was responsible for decreasing the antioxidant activity of OSP, RR, and the flake products. A positive correlation was observed between the reduction of bioactive compounds and antioxidant capacity. Most bioactive compounds are heat sensitive, which influences their antioxidant activity. A previous study reported that an increase in temperature accelerated the initiation of oxidation, preventing antioxidant compounds from working optimally [51]. The bioactive compounds were degraded, experiencing structural changes, and wholly transformed into inactive substances. Nevertheless, due to the complex nature of antioxidant compounds in plants, their thermal stability varies. Some compounds such as pro-catechuic acid, p-coumaric acid, and ferulic acid have high thermal stability, which facilitates their extraction and antioxidant activity [52]. The heat treatment process assists in the release of such compounds without affecting their activity. Moreover, the flake products exhibited lower antioxidant activity than the raw or boiled products. Based on the percentage values of DPPH and Superoxide radical scavenging capacity and the content of Fe [II] formed, flakes containing only OSP or RR had lower antioxidant activity. Interestingly, the combination of OSP and RR increased the antioxidant activity, probably due to the synergistic effect of bioactive compounds from OSP and RR [53]. Even though the processing reduced the antioxidant activity in boiled samples and flake products, the remaining antioxidant activity was still considerably high. Therefore, boiled OSP, RR, and their flake products are a good source of bioactive compounds and antioxidants.

Regarding the physicochemical and sensory properties of the flakes, it was observed that the moisture content of flake products was mainly influenced by their composition. Starch is the dominant carbohydrate found in OSP and RR, and OSP has a lower amylose content compared to RR. According to Wang et al. [54], the amylose content of OSP is 18.71%, while Markus et al. [55] reported that RR has 23% amylose content. Amylose is a linear polymer of glucose, which forms starch. The higher the amylose content, the greater the moisture content of the dough due to a higher capability to absorb water. The absorbed water will promote dough gelatinization during heating. The water absorbed by the dough will then evaporate during the flaking process due to the network’s inability to entrap water during the pre-gelatinization heat treatment. The high flaking temperature will detach water from the matrix structure of the flakes, resulting in increased evaporation and a lower moisture content of the flake products.

The dietary fiber content of the flakes ranged between 9.47 and 13.86%, comparable to values commonly found in breakfast cereals such as corn flakes, rice, quinoa, millet, and amaranth flakes [56]. The proportion of RR affected the dietary fiber of flakes. The fiber content of flakes was also influenced by the heating and pressing processes. Heat treatment caused the degradation of the fiber matrix and the glycosidic bond. The degradation affected the solubility level of the fiber, i.e., the ratio between soluble and insoluble fiber, thus resulting in the reduction of total fiber in the product.

Water absorption index (WAI) is a physical property associated with the ability of flakes to absorb water molecules within a particular time. Absorbed water molecules could be bound or detained in matrix pores of flakes. The water absorption index is crucial because it is associated with the quality of the flakes. Consumers can experience the crispy and crunchy sensation of the flakes after soaking in milk or water. In contrast, the higher WAI is interrelated with the unwanted soggy texture of flakes. The WAI is influenced by the porosity of the matrix on flakes, thickness, and hygroscopicity of flakes. In addition, the presence of fiber and protein could assist the flakes in absorbing water into their structure. The result shows that increasing the proportion of RR reduces the WAI due to a decrease in hygroscopicity. OSP is a rich source of sugar; thus, it has higher hygroscopicity compared to RR. In addition, the presence of RR affects the network construction of flakes by inhibiting the formation of the starch–protein structure, which could entrap gas. The compact structure created by RR starch inhibits water absorption into the matrix of the product. On the other hand, RR has higher fiber and protein contents, which help improve water absorption [57]. Moreover, the suitable fragmentation of the amylose and amylopectin chain in sweet potato could also affect the water absorption capacity. The heating processes such as roasting, flaking, and extrusion will induce starch fragmentation with sufficient water. The gelatinization process converts starch to a digestible material and plays a vital role in determining the structural properties of flakes and their ability to absorb moisture. The presence of RR in the flake dough disturbs the composition of starch, thus inhibiting the flakes from forming a porous structure and affecting their water absorption capacity. 

Fracturability is a physical property related to deformation conditions when a specific maximum force is applied. A higher fracturability value represents the ability of food products to maintain their structure when force is applied. According to Table 2, flakes with 100% OSP have a higher fracturability value compared to others, as the homogenous matrix of starch, protein, and fiber in OSP enables the interaction between the matrix and water molecules, leading to the firm, sturdy and rigid texture of flakes. The rigid texture is related to the evolution phase of starch from amorphous conditions, which indicates complete disorganization of the crystalline structure of starch. Increasing the proportion of RR in the flakes reduces the fracturability value. The mixture of OSP and RR decreases the rigidity of the flakes due to the different structural properties of the two samples, creating flakes that are susceptible to fracture. Crispness is a complex texture attribute because it comprises a combination of sensory analysis, acoustical procedure, and instrumental analysis. The instrumental analysis revealed that flakes with OSP to RR ratios of 100:0 and 0:100 had higher crispness values than others. Similar to fracturability, the crispness value decreased with an increasing RR proportion in the flakes. The mixture of ingredients with different structural properties can affect the crispness value of flakes.

The color profile shown in Table 3 revealed that the color of flakes was affected by the pigments in the raw materials used for their production. The red color of RR is associated with anthocyanins found in its bran layer. The yellowness of OSP is linked to carotenoids, primarily β-carotene. Previous research reported that carotenoids are easily oxidized and undergo color degradation due to thermal treatment [58]. The hue results showed a flake color range between yellow and red. The color of flakes was also affected by the Maillard reaction product. The higher the Maillard reaction product, the darker the appearance of flakes. 

The sensory analysis involved a preference test of the color, taste, crispness, and mouthfeel. Flakes containing 100% OSP had the lowest color preference score, associated with the orange appearance, and the lowest brightness score. Most panelists perceived the dark orange color as less fresh, less attractive, and less tasty. The color preference was increased with the addition of RR, which also helped improve the product lightness and redness values. The panelists were mostly in favor of flakes that appeared brighter and reddish. The higher brightness level is attributed to the white endosperm color of RR, while the redness is related to the anthocyanins in the bran of RR. The mouthfeel preference of flakes is associated with the water absorption index. The higher the ability of flakes to absorb water, the greater the plasticizing effect due to the presence of more hydrophilic components such as the phosphate monoester found in sweet potato starch [59]. The panelists generally preferred flakes with soft mouthfeel. The taste and crispness preferences for OSP- and RR-based flakes were in the range of “indifferent” and “slightly likes”. Increasing the RR proportion in flakes decreases the bitterness intensity and increases the savory taste. The perception of savory taste is generally influenced by the moisture content and the flavor of RR. Niu et al. [60] suggests that sweet potato with a low dry matter content has a bitter taste, and increasing the dry matter reduces the bitterness. On the other hand, a decrease in invertase activity may support the bitter aftertaste of the sweet potato.

This research successfully monitored the changes of the bioactive compound and antioxidant activity of OSP and RR in their native form and in the flake products. It can be observed that individual compounds acted differently to processing methods. Therefore, it can be suggested that research on the development of functional foods should address the products ready to be consumed instead of solely focusing on the raw materials due to the changes that take place during the transformation. This approach should be implemented for other potential materials rich in bioactive compounds. Moreover, further consideration of the bioaccessibility and bioavailability that are affected by digestion and absorption in the human metabolism system should also be considered [61].

## 5. Conclusions

OSP and RR are rich sources of bioactive compounds, especially β-carotene, for OSP, and phenolic compounds and anthocyanins, for RR. The boiling process significantly decreased most of the bioactive compounds, except tocopherols and α-carotene. The level of bioactive compounds in the flake products was dependent on the proportion of OSP and RR. Heat treatment resulted in a decrease in antioxidant activity, even though the remaining activity was still considerably high. The mixture of OSP and RR can produce flakes with low moisture and high fiber contents. The optimum flake water absorption index, fracturability, and crispness were obtained by combining 40% OSP and 60% RR. Moreover, the ratio of OSP and RR influenced the color and sensory preferences of the panelists.

## Figures and Tables

**Table 1:** Bioactive compounds of orange sweet potato (OSP), red rice (RR) and the flake products.

	OSP		RR		Proportions of OSP and RR in Flakes					
	Raw	Cooked	Raw	Cooked	100:0	80:20	60:40	40:60	20:80	0:100
Phenolic (mg GAE/100 g DW)	110.68 ± 18.3 ^a^	65.21± 7.3 ^b^	301.89 ± 24.86 ^a^	152.91 ± 28.92 ^b^	77.46 ± 8,28 ^a^	97.34 ± 8.79 ^b^	102.03 ± 11.65 ^c^	131.79 ± 10.93 ^d^	146.09 ± 15.64 ^e^	162.40 ± 21.54 ^f^
Anthocyanin (mg/100 g DW)	nd	nd	8,81± 0.05 ^a^	8.64± 0.08 ^a^	nd	nd	1.74 ± 0.06 ^c^	2.09 ± 0.05 ^d^	3.73 ± 0.07 ^e^	5.81 ± 0.04 ^f^
β-carotene (µg/g)	278.58 ± 31.5 ^a^	134.17± 17.2 ^b^	13.17 ± 2.62 ^a^	7.37 ± 0.5 ^b^	48,83 ± 3,31 ^a^	36.27 ± 3.01 ^b^	25.77 ± 3.45 ^c^	27.23 ± 2.72 ^d^	15.69 ± 2.21 ^e^	3.12 ± 0.66 ^f^
α-carotene (µg/g)	19.57 ± 1.8 ^a^	23.83 ± 1.6 ^b^	5.53 ± 1.4 ^a^	11.66 ± 1.5 ^b^	15.61 ± 1.44 ^a^	11.82 ± 3.11 ^b^	5.31 ± 1.59 ^c^	2.53 ± 0.87 ^d^	nd	nd
β-cryptoxanthin (µg/g)	4.83 ± 0.2 ^a^	4.48± 0.8 ^a^	3.67 ± 2.15 ^a^	3.96 ± 1.9 ^a^	2.64 ± 0,05 ^a^	2.81 ± 0.13 ^b^	2.97 ± 0.08	2.78 ± 0.12 ^c^	2.77 ± 0.25 ^c^	2.81 ± 0.16 ^b,c^
Lutein (µg/g)	3.77 ± 0.8 ^a^	3.81 ± 0.7 ^a^	2.16 ± 0.8 ^a^	1.82 ± 0.5 ^b^	nd	nd	nd	nd	nd	nd
α-tocopherol (µg/g)	13,23 ± 1.1 ^a^	15.11 ± 0,5 ^b^	34.08 ± 2.2 ^a^	57.65 ± 2.1 ^b^	4.58 ± 0.73 ^a^	7.34 ± 1.49 ^b^	10.51 ± 1.27 ^c^	12.45 ± 1.21 ^c^	16.82 ± 0.52 ^e^	18.31 ± 0.77 ^f^
γ-tocopherol (µg/g)	2.40 ± 0,2 ^a^	5.38± 0.05 ^b^	29.27 ± 2.4 ^a^	40,11 ± 1.8 ^b^	nd	nd	3.38 ± 1.22 ^c^	6.71 ± 1.19 ^d^	8.06 ± 0.98 ^e^	12.15 ± 0.73 ^f^

Data are presented as mean ± standard deviation. Different superscript letters (a–f) denote significantly different values according to Duncan’s test (*p* < 0.05). Comparison was made within each category (OSP, RR, and Flakes).

**Table 2:** The physicochemical properties of flakes produced from different ratios of Orange Sweet Potato (OSP), Red Rice (RR).

	Proportions of OSP and RR in Flakes					
	100:0	80:20	60:40	40:60	20:80	0:100
Moisture content (%)	5.71 ± 0,07 ^a^	5.31 ± 0.10 ^b^	5.09 ± 0.06 ^c^	4.87 ± 0.01 ^d^	4.43 ± 0.03 ^e^	4.25 ± 0.03 ^f^
Dietary fiber (%)	9.47 ± 0,01 ^a^	9.9 ± 0.02 ^b^	10.9 ± 0.05 ^c^	11.63 ± 0.34 ^d^	12.73 ± 0.26 ^e^	13.86 ± 0.73 ^f^
Water absorption index	1.69 ± 0,03 ^a^	1.14 ± 0.02 ^b^	1.06 ± 0.03 ^c^	0.96 ± 0.03 ^d^	1.09 ± 0.03 ^b,c^	1.12 ± 0.02 ^b,c^
Fracturability	8.48 ± 0,09 ^a^	5.35 ± 0.85 ^b^	3.34 ± 0.34 ^c^	2.27 ± 0.04 ^d^	3.17 ± 0.09 ^e^	4.64 ± 0.12 ^f^
Crispness	3.9 ± 0,03 ^a^	2.4 ± 0.02 ^b^	1.5 ± 0.02 ^c^	1.9 ± 0.03 ^d^	3.21 ± 0.05 ^e^	3.7 ± 0.03 ^f^

Data are presented as mean ± standard deviation. Different superscript letters (a–f) denote significantly different values according to Duncan’s test (*p* < 0.05). Comparison was made within each row.

**Table 3:** Color profiles of flakes produced from different ratios of orange sweet potato (OSP), red rice (RR).

	Proportions of OSP and RR in Flakes					
	100:0	80:20	60:40	40:60	20:80	0:100
L*	44.0 ± 0.1	47.3 ± 0.2	51.5 ± 0.1	52.7 ± 0.4	51.8 ± 0.2	51.8 ± 0.7
a*	8.2 ± 0.3	8,5 ± 0.3	8.9 ± 0.4	9.4 ± 0.4	10.2 ± 0.6	10.8 ± 0.4
b*	16.5 ± 0.3	14.7 ± 0.2	13.4 ± 0.2	10.3 ± 0.2	9.0 ± 0.4	5.9 ± 0.4
^o^h	63.574	59.9622	56.4087	47.6158	41.4237	28.6476
C	18.47	16.9685	16.0703	13.898	13.56	12.3145

L*: Lightness; a*: redness; b*: yellowness; ^o^h: ^o^hue; C: Chroma.

**Table 4:** Preference test of flakes produced from different ratios of orange sweet potato (OSP), red rice (RR).

	Proportions of OSP and RR in Flakes					
	100:0	80:20	60:40	40:60	20:80	0:100
Color	3.35 ± 1.42 ^a^	4.40 ± 1.22 ^b^	5.14 ± 1.12 ^c^	4.93 ± 1.21 ^c^	4.89 ± 1.30 ^c^	4.30 ± 1.12 ^b^
Taste	4.43 ± 1.34 ^a^	4.69 ± 1.28 ^ab^	5.08 ± 1.18 ^c^	5.09 ± 1.20 ^c^	5.05 ± 1.26 ^bc^	4.72 ± 1.29 ^abc^
Crispness	4.76 ± 1.16 ^b^	4.85 ± 1.04 ^b^	4.76 ± 1.40 ^b^	4.08 ± 1.17 ^a^	4.96 ± 1.07 ^b^	5.00 ± 1.04 ^b^
Mouthfeel	5.41 ± 0.91 ^d^	5.04 ± 1.18 ^c^	4.84 ± 1.31 ^bc^	3.91 ± 1.59 ^a^	4.66 ± 1.32 ^b^	5.05 ± 1.03 ^c^

Data are presented as mean ± standard deviation. Different superscript letters (a–d) denote significantly different values according to Duncan’s test (*p* < 0.05). Comparison was made within each row.

## Data Availability

Data is contained within the article.
